# How can basic research on spatial cognition enhance the visual accessibility of architecture for people with low vision?

**DOI:** 10.1186/s41235-020-00265-y

**Published:** 2021-01-07

**Authors:** Sarah H. Creem-Regehr, Erica M. Barhorst-Cates, Margaret R. Tarampi, Kristina M. Rand, Gordon E. Legge

**Affiliations:** 1 Department of Psychology, University of Utah, Salt Lake City, UT USA; 2 Moss Rehabilitation Research Institute, Elkins Park, PA USA; 3 Department of Psychology, University of Hartford, West Hartford, CT USA; 4 Department of Psychology, University of Minnesota, Minneapolis, MN USA

**Keywords:** Low vision, Space perception, Spatial cognition, Design

## Abstract

People with visual impairment often rely on their residual vision when interacting with their spatial environments. The goal of visual accessibility is to design spaces that allow for safe travel for the large and growing population of people who have uncorrectable vision loss, enabling full participation in modern society. This paper defines the functional challenges in perception and spatial cognition with restricted visual information and reviews a body of empirical work on low vision perception of spaces on both local and global navigational scales. We evaluate how the results of this work can provide insights into the complex problem that architects face in the design of visually accessible spaces.

## Significance

Architects and designers face the challenge of creating spaces that are accessible for all users, following the principles of Universal Design. The proportion of the population who have uncorrectable visual impairment is large and growing, and most of these individuals rely on their residual vision to travel within spaces. Thus, designing for visual accessibility is a significant practical problem that should be informed by research on visual perception and spatial cognition. The work discussed in this paper presents an empirical approach to identifying when and how visual information is used to perceive and act on local and global features of spaces under severely restricted vision. These basic research approaches have the potential to inform design decisions that could improve the health and well-being of people with low vision and extend more broadly to enhance safety and effective use of designed spaces by all people.

## Introduction

Millions of people across the world have *low vision*, defined as significant uncorrectable visual impairment that impacts essential everyday tasks. Notably, people with low vision have useful residual visual capabilities and often rely on vision as a primary source of information guiding perception and action within their environments. Given this reliance on vision, an important goal in the design of spaces is to increase *visual accessibility*, to enable the design of environments that support safe and efficient travel for those with visual impairment. Visual accessibility is necessary for full participation within our society, as the ability to travel effectively through one’s environment is critical for independence in accomplishing daily tasks. Limitations in independent mobility due to vision loss lead to debilitating consequences related to quality of life, such as social isolation, reduced opportunities for education and employment, and economic disadvantage.

The goal of this paper is to evaluate how basic research in space perception and spatial cognition can inform the practical design of architectural spaces to improve visual accessibility for people with low vision. First, we provide a background on the prevalence of low vision and the “dimensions” of low vision (reduced acuity, reduced contrast sensitivity, and visual field loss) that are likely to affect space perception and spatial cognition. We discuss the possible effects of reduced visual information on the recruitment of other sensory modalities and the motor system for gathering spatial information, as well as the impact of navigation with low vision on higher-level attention and memory processes. Second, we provide a critical review of studies of low vision concerned with perception on local and global spatial scales, a distinction important to theories of spatial representation and navigation (Ekstrom and Isham 2017; Montello 1993; Wolbers and Wiener 2014). Third, we review the concept of Universal Design and the need to design for visual accessibility analogous to more familiar approaches of designing for physical accessibility. We consider the challenges that architects and lighting designers face in working at multiple scales of space and argue that an understanding of spatial processing with reduced visual information could inform design decisions.

## Low vision: prevalence and functional consequences

Estimates of the prevalence of visual impairment vary depending on criteria used, but by all accounts, the number of people who have uncorrectable vision loss is startling. About 441.5 million people are visually impaired worldwide, but only a small percentage (about 8%) have total blindness (Bourne et al. 2017) and most are characterized as having *low vision*. People with low vision have some remaining functional vision and use their residual visual capabilities for many tasks, including reading, object recognition, mobility, and navigation. Low vision is characterized as visual acuity less than 20/40 or a visual field of less than 20°. Clinical diagnosis of severe to profound visual impairment is often defined as 20/200 to 20/1000. In the USA, the statutory definition for legal blindness is defined as best-corrected visual acuity of 20/200 in the better eye or a visual field of no more than 20° (Giudice 2018). Recent estimates in the USA show about 5.7 million Americans with uncorrectable impaired vision, and this number is projected to double by 2050 (Chan et al. 2018). The number of adults in the USA at risk for vision loss (as defined by factors of older age, diabetes, eye disease) increased by 28 million from 2002 to 2017 to a total of 93 million adults at risk (Saydah et al. 2020). This high prevalence and increased risk for low vision should be of significant concern, particularly as associated limitations in the ability and motivation to travel independently are highly related to increased social isolation, depression, and economic disadvantages (Giudice 2018; Marston and Golledge 2003; Nyman et al. 2010).

**Fig. 1:**
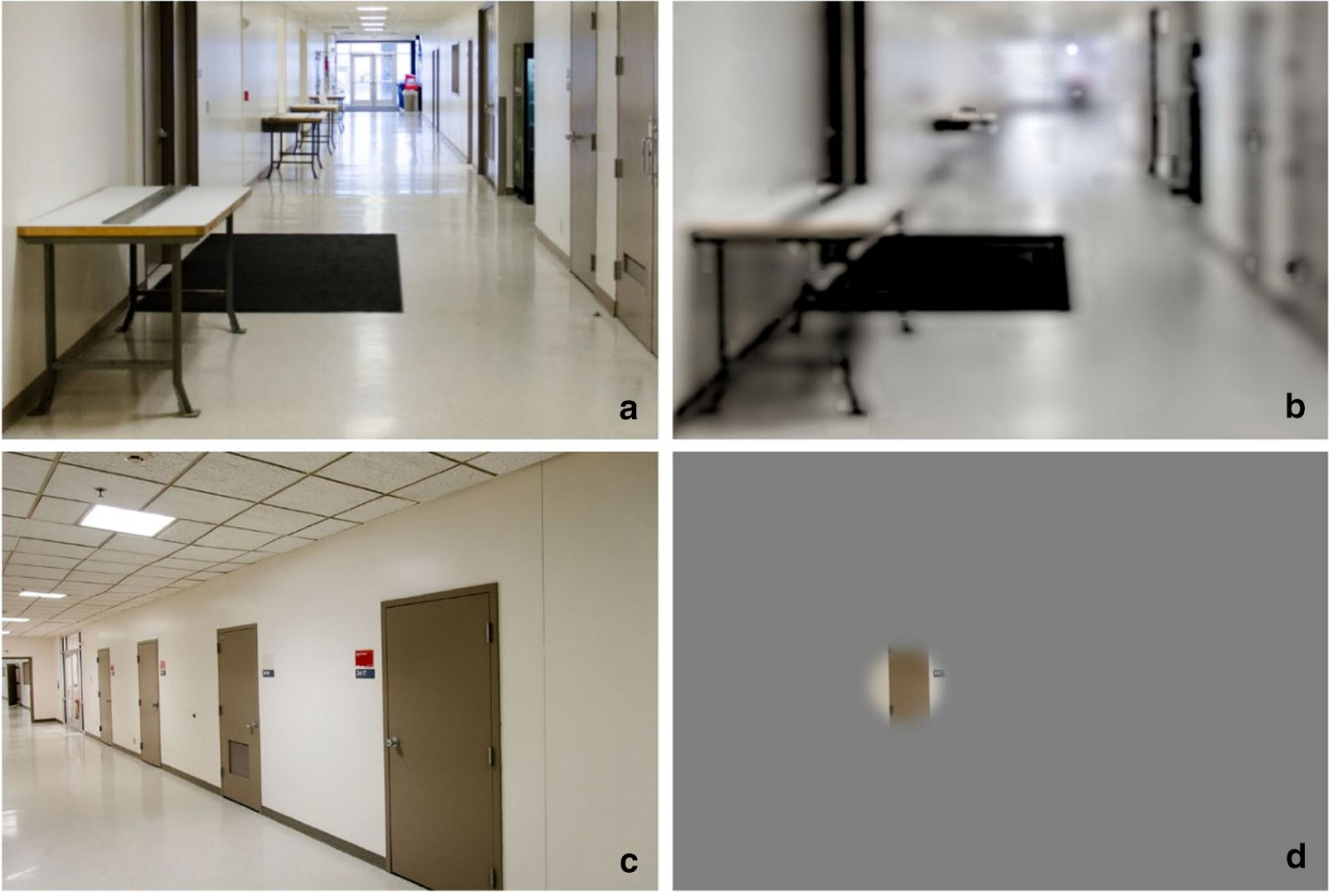
Hallway scenes with normal vision and simulated low vision show possible effects on visibility and use of environmental features for spatial behavior. Photographs by William B. Thompson

While the dimensions of low vision are often reported clinically in terms of acuity and contrast sensitivity levels and extent of field of view,^1^ in the work described here we attempt to demonstrate the functional relationship between characterizations of vision loss and spatial behavior. Functioning actively within built spaces relies on the ability to detect and identify environmental geometry such as steps, pillars, or benches so that they do not become mobility hazards. These environmental features also serve a role in providing spatial context such as frames of reference or landmarks to aid in spatial updating, keeping track of one’s current location and orientation in space while moving. Figure 1 provides illustrations of the effect of reduced acuity and contrast sensitivity and reduced peripheral field of view on visibility and use of environmental features. The top two images show a hallway scene with normal acuity (a) and under a simulated acuity of logMAR 1.1 (20/250 Snellen) and Pelli-Robson score of 1.0 (b) (Thompson et al. 2017). In the low vision image, the near table is still recognizable, the mid-distance table is detectable as some sort of feature but is not recognizable, and the more distant tables are essentially invisible. The bottom pair of images shows a normal view of a hallway (c) and a simulation of peripheral field loss (d), with a remaining field of 7.5°. While central field acuity and contrast sensitivity are unaffected, tasks such as finding the second door on the right are made much more difficult.

One primary approach to assess the impact of low vision on these components of space perception and navigation has been to artificially reduce acuity, contrast sensitivity, or visual field in those with normal vision and test perception and spatial cognition in controlled but real-world laboratory settings. We use the term *simulated low vision* to describe these artificial reductions, but it is not our intention to convey a specific pathology or assume an accurate representation of the subjective experience of low vision. These studies create viewing restrictions with goggles fitted with occlusion foils or theatrical lighting filters. Much of the experimental work described in this paper falls within the range of severe to profound simulated low vision. Admittedly, simulations using artificial restrictions with normally sighted people do not reproduce behavioral adaptations to vision loss or capture the wide individual variability in low-vision conditions. However, as demonstrated in this review, low vision simulations are a valuable approach because they provide a controlled and less-variable way to assess effects of reductions in visibility of environmental features. This review focuses on the generic effects of reduced spatial resolution, contrast, and field on perceptual interpretation and spatial cognition. While there may be some interactions between specific diagnostic categories, such as glaucoma or macular degeneration, and the cognitive and perceptual factors we are considering, we expect that similar cognitive and perceptual limitations are shared quite broadly across low-vision conditions. We also review some work testing people with actual low vision, showing qualitatively similar effects on perception and recognition of features as found with the low vision simulations.

Predictions about abilities to identify and use environmental features for safe and efficient travel can be informed by the limitations of visual information. For example, those with reduced acuity and contrast sensitivity should have more stringent requirements for angular size of objects and their contrast with surrounding surfaces in order to detect and recognize objects. Reduced acuity and contrast sensitivity should also impact the information that can be used for perceiving scale and distance, such as reliance on high-contrast boundaries rather than high-resolution textures. These features serve as the building blocks for spatial updating and higher-level spatial representations of one’s environment, so we also expect to see influences of low vision on spatial cognition. For example, many models of navigation emphasize visual landmarks (e.g., Chan et al. 2012; Chrastil and Warren 2015; Ekstrom 2015; Epstein and Vass 2014) and environmental geometry (Marchette et al. 2014; Mou and McNamara 2002) as providing frames of reference for spatial learning. Here, in addition to reduced acuity and contrast sensitivity, field of view should also play a role, as it should be more difficult to perceive the scale and shape of large-scale environmental geometry or encode global configurations when experienced in multiple restricted visual snapshots (Fortenbaugh et al. 2007, 2008; Kelly et al. 2008; Sturz et al. 2013). Importantly, landmark recognition, self-localization, and formation and use of long-term spatial knowledge all involve some amount of attentional resources (Lindberg and Gärling 1982), and low vision increases these attentional demands (Pigeon and Marin-Lamellet 2015). Low-vision mobility itself requires attentional resources which compete with the attention needed to form spatial memories (Rand et al. 2015). We also consider the important role of non-visual body-based information (specifically proprioceptive and vestibular) for spatial updating and spatial learning, that is relied on by both individuals who are normally sighted and those with visual impairment (Giudice 2018). Much of the work reviewed here does not focus on auditory or tactile sensory input, although other work suggests that spatialized sound (Giudice et al. 2008) and tactile-audio interfaces (Giudice and Palani 2014) have the potential to support and enhance spatial navigation performance for people with vision loss.

## Impact of low vision on space perception: use of local features

**Fig. 2:**
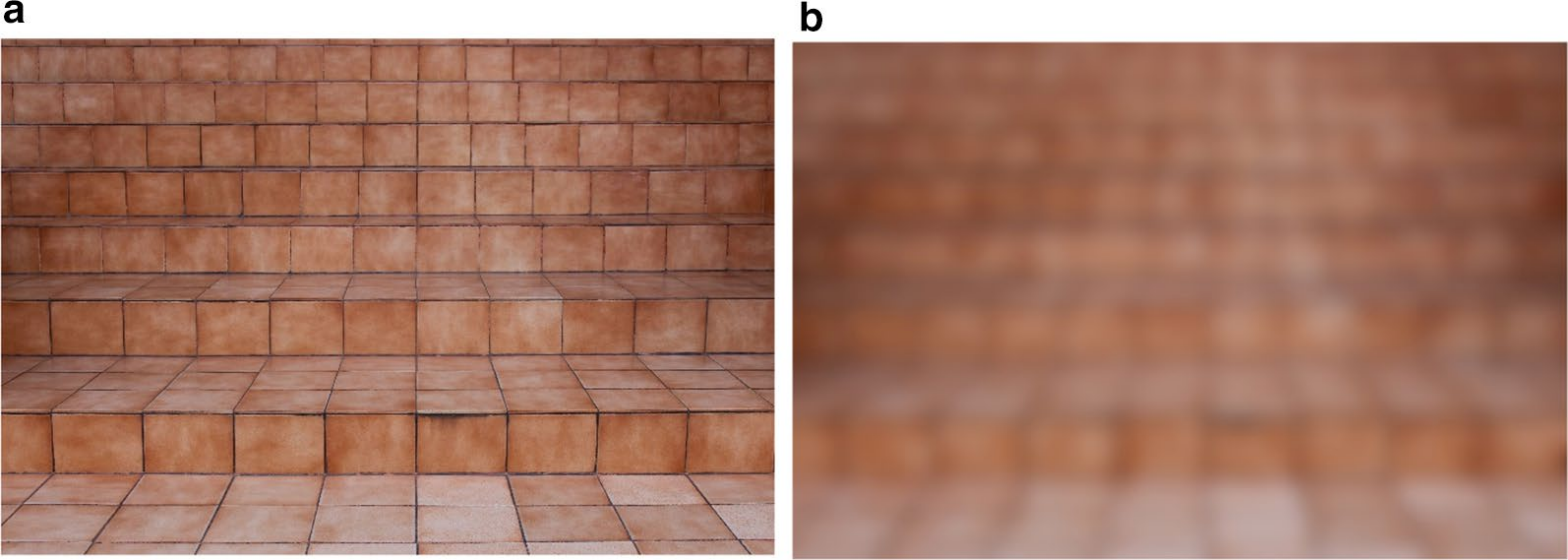
Steps viewed with normal vision (**a**) as compared to simulated degraded acuity and contrast sensitivity (**b**) demonstrating loss of detail and texture gradient of steps

Much of the early research on perception of environmental features in the context of low vision was focused on obstacle avoidance while moving through spaces. This work suggested that visual field loss was a major contributor to safely avoiding visual hazards during locomotion, whereas acuity and contrast sensitivity were less important (e.g., Kuyk et al. 1998; Long et al. 1990; Marron and Bailey 1982, Pelli 1987). While essential for mobility, obstacle avoidance during walking relies on dynamic cues for distance and self-motion and, as a task, may not reveal the critical contribution of acuity and contrast needed for perception of environmental features from a distance (Ludt and Goodrich 2002). From static viewpoints or farther distances, irregularities of ground plane surfaces such as steps and ramps, as well as environmental objects such as benches, posts, and signs may not be visible given low contrast with surrounding surfaces or smaller angular size. Reduced acuity and contrast can affect familiar size cues and perspective-based information used for perceiving distance and scale by reducing high-frequency detail and texture gradients (see Fig. 2). These surfaces and objects can become hazards when not detected, recognized, or localized, and their visibility is important to consider when designing for visual accessibility.

**Fig. 3:**
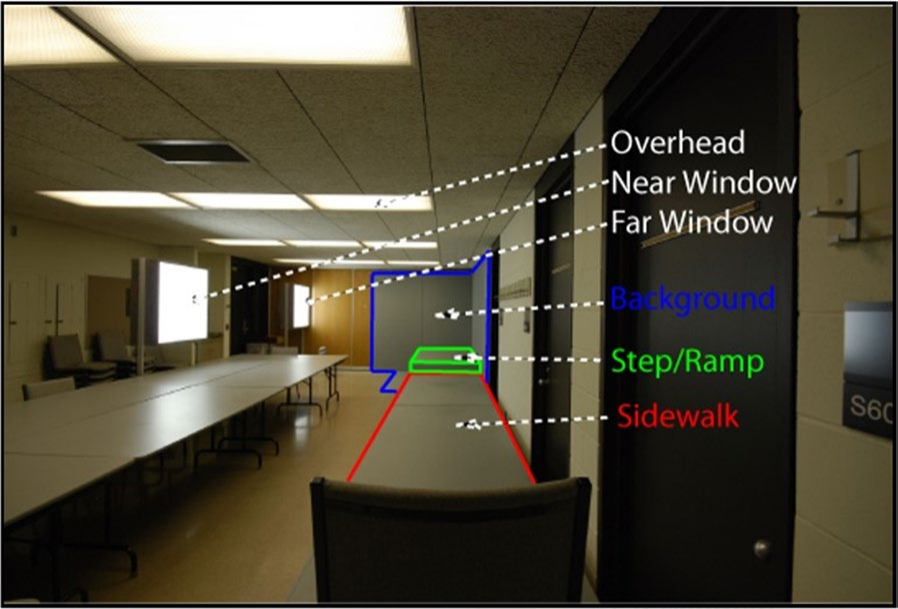
The constructed sidewalk and room used for the steps and ramps studies. Adapted from Legge et al. (2010)

**Fig. 4:**
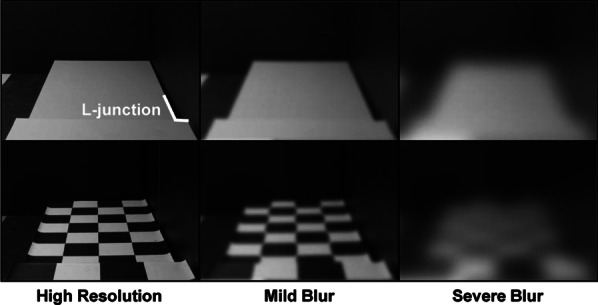
The step-down target used in Bochsler et al. (2012). Reprinted with permission from Wolters Kluwer Health, Inc. The Creative Commons license does not apply to this content

To begin to understand the impact of visibility of ground-plane irregularities on visual accessibility, Legge et al. (2010) created a long sidewalk inside of an indoor windowless classroom that could be interrupted by a step or ramp, as shown in Fig. 3. The goal was to test detection and recognition of these steps and ramps in the context of manipulations of lighting direction, target-background contrast, and viewing distance, at different levels of simulated acuity and contrast sensitivity loss created through restricted viewing goggles (referred to as “blur”), as these were predicted to influence the visibility of the cues used to distinguish the environmental feature (see Table 1 for details about local cue studies). Several take-home messages emerged. Steps up were more visible than steps down, and visibility could be helped by enhancing contrast between the riser and contiguous surface with directional lighting. Local image features such as discontinuities in edge contours of a walkway at a step boundary were sources of information highly dependent on viewing distance and contrast (see L-junction in Fig. 4). Finally, viewers used the height of the end of the walkway in their visual field to distinguish between a ramp up and a ramp down, showing that the cue of height in the picture plane may be more reliable than local ground surface cues to those with blurred vision because it is less dependent on acuity. Further studies using the same paradigm asked whether providing a high contrast checkerboard texture on the sidewalk would facilitate recognition of the environmental geometry under blur viewing conditions (Bochsler et al. 2012). Surprisingly, presence of the surface texture detracted from accuracy in the severe blur condition. Apparently, the transition contrast cue shown to be used to recognize a step up was masked by the high-contrast texture edges from the checkerboard pattern. Similarly, the texture under severe blur appears to mask the L-junction that could be used as a cue to step down (see Fig. 4). People with moderate to severe low vision also participated in the same ramps and steps paradigm (Bochsler et al. 2013). Overall, they outperformed the normally sighted participants with simulated low vision from Legge et al. (2010), but the effects of distance, target type, and locomotion were qualitatively similar for the low vision and normal vision participants. Furthermore, environmental objects themselves can become hazards if they are not detected or recognized. Kallie et al. (2012) identified advantages in object identification for specific shapes and colors that depended on lighting conditions, as well as for larger and closer objects.

**Table 1:** Summary of task design, manipulations, results and implications for the *Local Features* papers included in the review

Local features					
Citation	Low vision type	Task paradigm	Main manipulations	Results	Implications for design
Bochsler et al. (2012)	Blur: 2 severity levels Mild: P–R = 0.8 logMAR = 0.88 (20/152) Severe: P–R = 0.6 logMAR = 1.65 (20/884)	Viewed and identified steps up or down, ramps up or down, or flat surfaces	Distance: 1.52, 3.05, and 6.10 m Blur severity Locomotion versus static viewing High-contrast checkerboard texture versus uniform gray surface	1. Poorer performance with textured compared to uniform surface with severe blur 2. Locomotion improved accuracy over static viewing	Avoid high contrast texture on ground surfaces Designing for active locomotion could facilitate feature recognition
Bochsler et al. (2013)	LV individuals (variety of types) Acuity range from logMAR 0.98 to 2.18 (20/191–20/3000) Field loss range of peripheral, central, or both	Viewed and identified steps up or down, ramps up or down, or flat surfaces	Distance: 1.52, 3.05, and 6.10 m Lighting: near and far Target-background contrast Locomotion versus static viewing	1. People with LV outperformed those with simulated LV and were not strongly affected by target-background contrast 2. Similar to simulated LV, people with LV showed better identification for step-up compared to step-down and benefited from closer distances and locomotion	Designing for active locomotion could facilitate feature recognition
Kallie et al. (2012)	Blur (severe) P–R = 0.6 Snellen 20/900	Viewed boxes and cylinders (2–6 ft) Detection, confidence for detection, shape, and height	Distance: 3.05, 5.18, 7.32 m Lighting: overhead or window Color: White, gray Object height: short, medium, tall Shape: box, cylinder	1. Cylinders were easier to identify compared to boxes 2. Advantage for color (white vs gray) depended on the lighting 3. Better performance resulted with larger and closer objects	Consider potential interactions of color, object shape, and lighting Overhead and window lighting may not differentially affect performance
Legge et al. (2010)	Blur: 2 severity levels Mild: P–R = 0.8 logMAR = 0.81–.85 Severe: P–R = 0.6 logMAR = 1.64–1.67	Viewed and identified steps up or down, ramps up or down, or flat surfaces Uniform gray surface with varying backgrounds	Distance: 1.52, 3.05, and 6.10 m Lighting: overhead, near window, far window Blur severity Background color: black or gray	1. Steps up were more visible than steps down 2. Local geometric cues for identification (e.g., shape of edge contours of a walkway in an image) were dependent on viewing distance and contrast 3. A cue for identifying a ramp was its elevation in the image	Visibility of steps down is of particular concern and may be enhanced by contrast between riser and contiguous surface, and directional lighting
Rand et al. (2011)	Blur (severe) logMAR = 1.60 (20/791)	Distance perception to targets	Visual horizon height (wall-floor boundary): actual or raised Distance: 3, 4.5, 6 m	1. When the “horizon” was raised, the angle of declination to the target increased, and viewers judged the distance to targets on the ground to be closer	Create high contrast between wall and floor to make the visual horizon salient
Rand et al. (2012)	Blur (severe) P–R = 0.46 logMAR = 1.51 (20/647)	Distance and size perception of targets on stands	Distance: 1.5, 2.7, 4 m Color of stands that the targets were placed on–created high or low contrast with ground plane Blur versus normal vision	1. Distance and size judgments were accurate to the targets presented on the visible black stands 2. When the stands were not visible (painted gray and viewed through blur goggles), participants overestimated size and distance to the target	Increase visibility of information for grounding targets when they are located above the ground surface
Tarampi et al. (2010)	Blur (severe) P–R = 0.36 logMAR = 1.53	Distance perception by blind walking to targets	Distance: 1.5, 3.1, 6 m Blur versus normal vision	1. Relatively accurate distance perception with blur, although with increased variability	Accuracy in distance estimation may be increased with salient visual horizon cues

*P–R* Pelli–Robson contrast sensitivity value, *LV* low vision, *Blur* simulated reduced acuity and contrast sensitivity, Snellen values are in parentheses

**Fig. 5:**
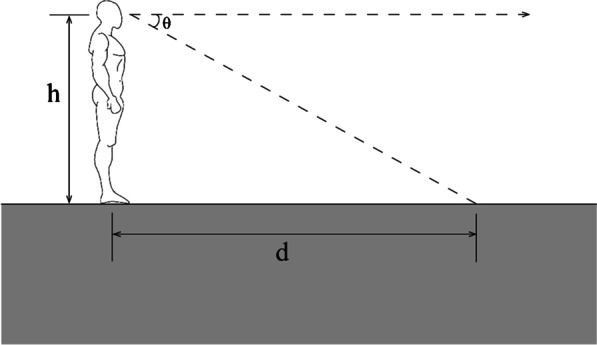
For a viewer standing on a ground plane, the distance (*d*) to locations on the ground can be computed using the horizon-distance relation (angle of declination), scaled by eye height (*h*): *d* = *h* cot *θ*. “Human body front and side” image by Nanoxyde licensed under CC BY-SA 3.0

The visibility of features is important not only for recognition of surfaces and objects, but also for spatial localization. Successful independent navigation depends on the ability to perceive distances and locations of environmental features, and update 3D representations of space with self-movement. Several studies have used low vision simulation paradigms to examine the perception of distance and size in room-sized environments. For example, in Tarampi et al. (2010), participants viewed targets in a large indoor room at distances up to 6 m and then walked directly or indirectly to targets while blindfolded. These “blind-walking” tasks are a type of visually directed action measure that indicates perceived distance. Indirect walking involves walking initially in one direction and then on a cue, turning and walking to the target location. Because preplanning motor strategies would be difficult in this unpredictable task, it is a good test of the viewer’s abilities to update their self-location with respect to the environment. Although targets were just barely visible, participants surprisingly showed accurate blind walking to these locations that was comparable to performance in normal vision conditions, revealing relatively intact distance perception, although with increased variability. One explanation for this relatively good performance despite severely degraded vision is that viewers used the visual horizon as a salient cue for judging distance. Sedgwick (1983) defined the horizon-distance relation or the use of angle of declination between the horizon and a target object as a mechanism for a viewer standing on the ground surface to recover absolute egocentric distance to a location on the ground (see Fig. 5).

**Fig. 6:**
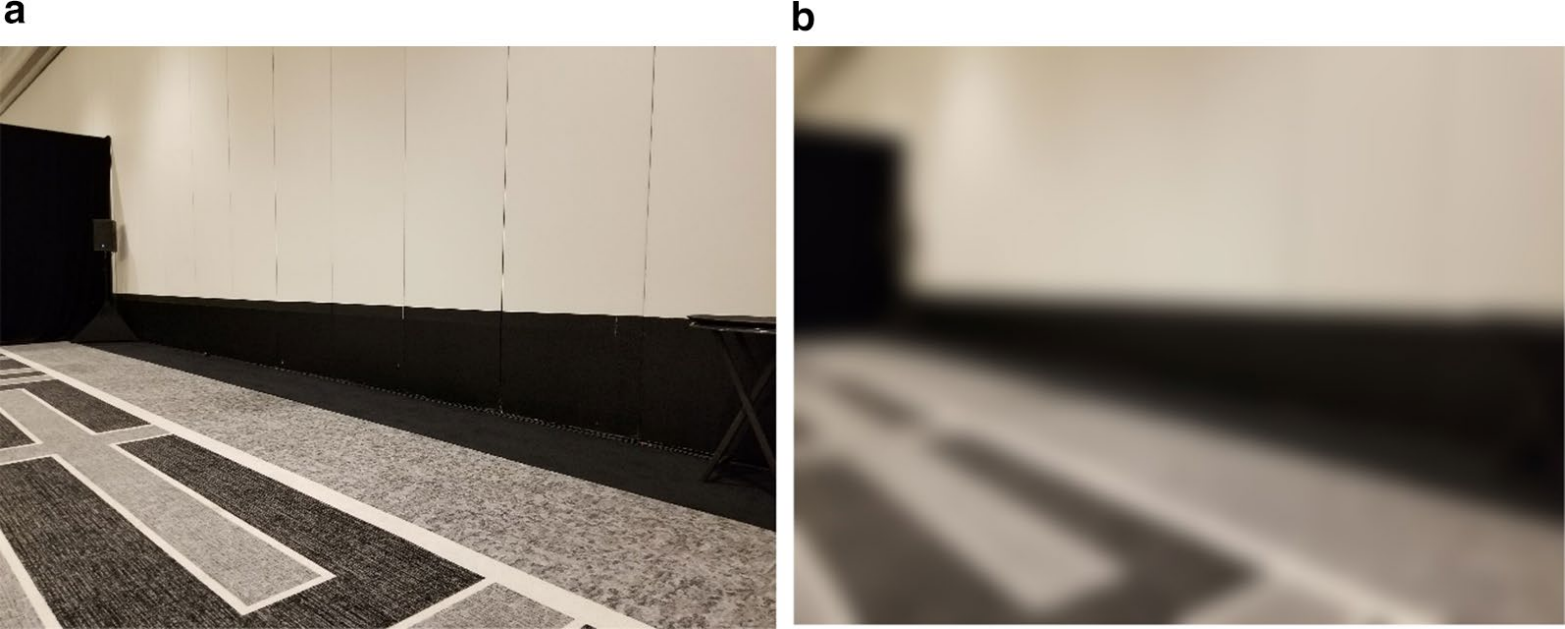
Conference Room at Loews Miami Beach Hotel in Miami Beach FL USA under normal vision (**a**) and simulated low vision (**b**). Photograph credit: Margaret Tarampi

When a viewer is standing on the ground, the distance to a location on the ground can be computed as a function of one’s eye height and the angle between the line of sight to the horizon and the line of sight to the object. For indoor spaces, the floor-wall boundary plays the role of the visible horizon. Rand et al. (2011) tested the role of the visual horizon as a cue in a low vision context by artificially manipulating the floor-wall boundary in a large classroom. Because viewers in this study wore blur goggles, it was possible to raise the visible boundary between the floor and wall by hanging material on the wall that matched the floor. When the “horizon” was raised, the angle of declination to the target increased, and as predicted, viewers judged the distance to targets on the ground to be closer. Figure 6 shows a real-world example of this effect. The black carpet on the floor and wall become indistinguishable under blurred viewing conditions, leading to a misperception of the visual horizon and potential errors in perceived distance.

**Fig. 7:**
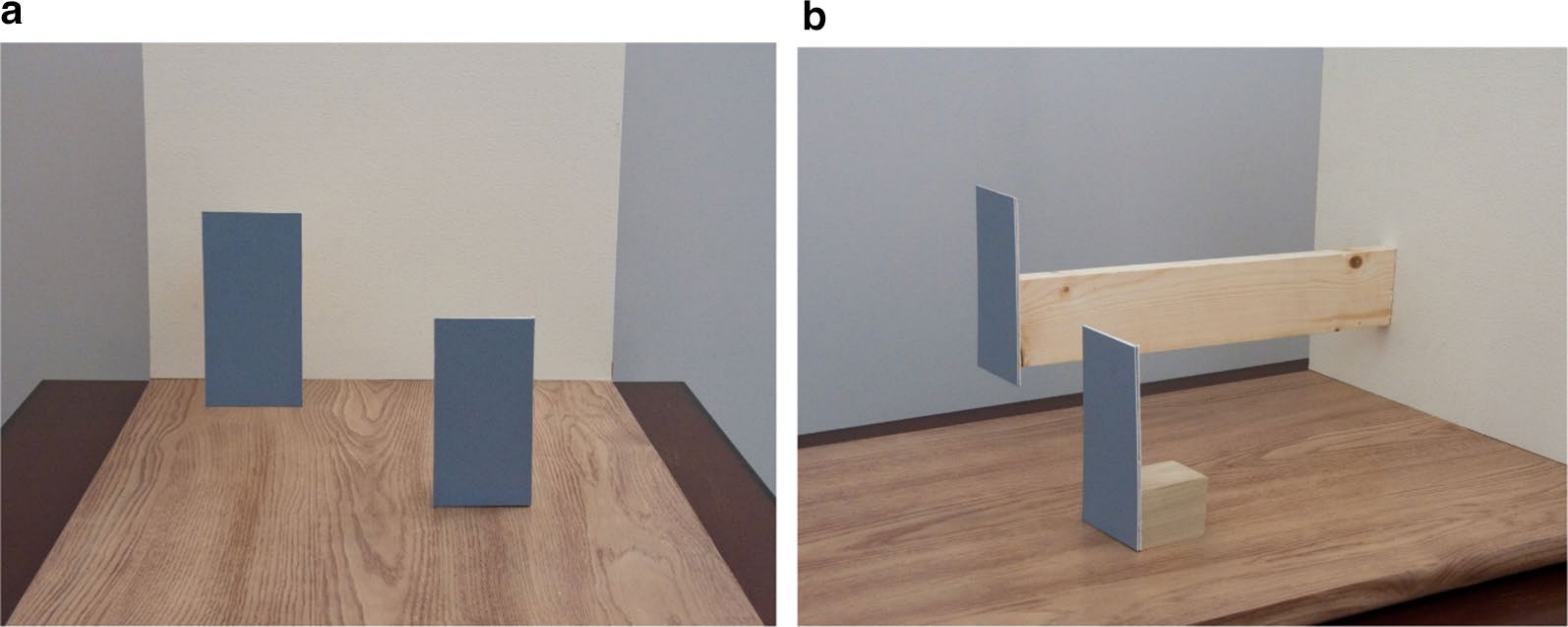
Images motivated by Gibson (1950) demonstration showing that in the absence of visual information specifying lack of contact with a support surface, a target that is off the surface is perceived to be on the surface but farther away (**a**). Image (**b**) shows the actual configuration in which both objects are the same distance from the camera and the left object is raised off the surface. Created by William B. Thompson

**Fig. 8:**
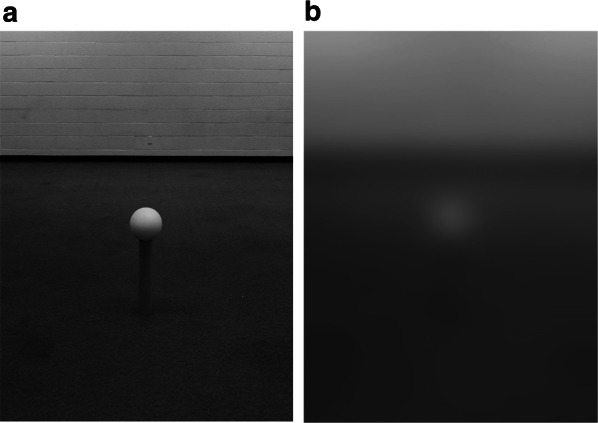
The gray stand is detectable with normal viewing (**a**), but undetectable under degraded vision (**b**). Viewing with blur goggles led to overestimation of distance and size of the target. Adapted from Rand et al. (2012) with permission from Brill

Further support for the importance of ground surface cues for distance in low vision comes from a study that manipulated the visual information for whether an object is in contact with the ground (Rand et al. 2012). Objects that we interact with often make contact with the ground plane, but that point of ground contact may not always be visible, particularly under blurred viewing conditions. For example, furniture may have small or transparent legs, or stands on which objects or signs rest may have low contrast with the ground surface. Gibson’s (1950) ground theory of perception and insightful demonstrations (see Fig. 7) posit that in the absence of cues to suggest that a target is off the ground, viewers will judge distance assuming that the target is in direct contact with the ground. Thus, a target that is off the ground, but assumed to be on the ground, will be perceived to be at a farther distance, consistent with the location on the ground plane that it occludes. In the context of visual accessibility, if the ground contact of an object is not visible, the misperception of the distance of that object could lead to critical collision hazards. Rand et al. (2012) tested whether manipulating the visibility of the ground-contact support for an object off the ground would lead to the predicted misperception of distance. Participants viewed targets placed on stands that were visible or not due to manipulation of high or low contrast between the stand and the ground plane and manipulations of simulated degraded acuity and contrast sensitivity (see Fig. 8). With normal viewing, the stands were visible and distance and size judgments to the targets were accurate. Viewing with blur goggles, the low-contrast gray stand became undetectable and distance and size of the target were overestimated, consistent with Gibson’s predictions of ground theory. These studies demonstrate the importance of the visibility of information for grounding targets when they are located above the ground surface. We will return to this finding in the discussion of implications for design.

## Impact of low vision on spatial cognition: global spatial features and locomotion

Thus far we have described the impact of low vision on the visibility of local features, demonstrating that severely blurred vision can impair visual perception of irregularities in surfaces such as ramps and steps, large-scale objects, and perception of distance to objects off the ground. These components are important to understanding spatial perception from static viewpoints at scales immediately surrounding the viewer that can be perceived without locomotion, defined as *vista* space (Montello 1993). However, much interaction with space entails actively traveling through it, requiring perception of distance traveled as well as memory for important landmarks, such as a bathroom or emergency exit. These global features of space are vital to consider for spatial navigation, a complex activity that involves perceptual, sensorimotor, and higher-level cognitive processes. There is a large literature on understanding navigation at both sensorimotor and higher cognitive levels in normally sighted people (for reviews see Chrastil and Warren 2012; Ekstrom et al. 2018) as well as in blind individuals (Giudice 2018; Loomis et al. 1993). Normally sighted individuals tend to rely on visual information when it is available and reliable (Zhao and Warren 2015), and studies with blind and blindfolded individuals sometimes reveal intact abilities to use non-visual information (Loomis et al. 1993; Mittelstaedt and Mittelstaedt 2001). However, the residual visual capacity in low vision raises important questions about how people navigate and remember important landmarks when visual information may be present but degraded, an area of research that has received much less attention in the literature.

The environments used to test the impact of low vision on navigation have ranged from simple one-legged paths, to 3 segment spatial updating tasks, to large-scale environments that vary in complexity from long narrow hallways to open environments requiring many turns (see Table 2 for details of global-feature studies). We generally see that low vision type and severity interact with task complexity to influence performance. Whereas the study of local features has focused primarily on the interaction of vision with reduced acuity with surface, geometry, and lighting conditions, examination of global features has extended to simulated peripheral field loss. Reduced peripheral field of view impacts use of global features in spatial cognition in numerous ways, including distance estimation (Fortenbaugh et al. 2007, 2008), perception of global configurations of spatial layout (Yamamoto and Philbeck 2013), encoding and use of environmental geometry as a frame of reference (Kelly et al. 2008; Sturz et al. 2013), and increasing cognitive load (Barhorst-Cates et al. 2016).

**Table 2:** Summary of task design, manipulations, results and implications for the *Global Features* papers included in the review

Global features					
Citation	Low vision type	Task paradigm	Main manipulations	Results	Implications for design
Barhorst-Cates et al. (2016)	Simulated FOV of 15°, 10°, 4°, or 0°	Real-world building-scale spatial learning paradigm Structured hallway environment	Vision condition (narrow vs. wide FOV)	1. Pointing accuracy to remembered targets was impaired at 4° and 0° FOV, but intact at 15° and 10° 2. Increased cognitive demands starting at 10° and lower	Design spaces to reduce mobility hazards and the need for safety monitoring
Barhorst-Cates et al. (2017)	Blur in an older adult sample P–R = 0.33 logMAR = 1.55 (20/708)	Real-world building-scale spatial learning paradigm Structured hallway environment	Physical guidance	1. Pointing accuracy to remembered targets increased with a physical guide 2. Reducing cognitive load can increase spatial learning with low vision	Designing a space to reduce mobility hazards/monitoring may be especially important for locations frequented by older adults
Barhorst-Cates et al. (2019)	Simulated FOV of 10°	Real-world building-scale spatial learning paradigm Complex structure; museum environment	Vision condition (narrow vs. wide FOV)	1. Pointing accuracy decreased in narrow compared to wide FOV 2. Greater attentional demands resulted in narrow versus wide FOV 3. Effect of reduced FOV may be greater in complex, open environments	Consider regularity and complexity in design of spaces
Barhorst-Cates et al. (2020)	Simulated FOV of 10°	Real-world building-scale spatial learning paradigm	Locomotion method (walking, wheelchair) Vision condition (narrow vs. wide FOV) Active versus passive search	1. Pointing accuracy to remembered targets was equivalent in walking versus wheelchair 2. Active search with narrow FOV demanded more attention than passive search	Make key locations salient without requiring significant visual search
Fortenbaugh et al. (2007)	Simulated FOV of 40°, 20°, 10°, or 0°	Spatial learning task in virtual environment Verbal and blind-walking distance estimates	Vision condition (FOV) Distance to targets (range from 2.7 to 11.1 m) Static versus walking during learning phase	1. Memory errors for target location increased with decreasing FOV 2. Greater errors at larger distances 3. Distance underestimated in all vision conditions when viewer was static	Given difficulty with far targets, provide frequent nearby signage that would be visible from multiple positions Use standard distance between important locations that is viewable from some minimum field-of-view (40°)
Fortenbaugh et al. (2008)	LV individuals (FOV loss)	Spatial learning task in virtual environment Replication in a real-world environment	FOV deficit	1. Memory errors for target location increased with decreasing FOV 2. Underestimated distances 3. Angular error not affected as much	Given difficulty with far targets, provide frequent nearby signage that would be visible from multiple positions Use standard distance between important locations that is viewable from some minimum field-of-view (40°)
Legge et al. (2016b)	Blind, LV, and normally sighted individuals	Distance and direction estimates using a path completion task	Level of sensory deficit	1. No difference between groups, suggesting that vision was not necessary for accurate spatial updating	Design implications for low vision may be more relevant for larger-scale spaces
Legge et al. (2016a)	Mild blur (20/135), severe blur (20/900), and narrow FOV (8°)	Distance and direction estimates using a path completion task Room size estimates	Room size vision condition Locomotion condition	1. Reduced vision conditions did not impair distance estimates compared to normal vision 2. Severe blur impaired direction estimates and room-size judgments 3. No difference between walking and wheelchair	Create high contrast between wall and floor to improve perception of room size
Rand et al. (2015)	Blur P–R = 0.76 logMAR = 1.44 (20/562)	Indoor navigation and spatial learning paradigm	Vision condition (Blur vs. normal vision) Physical guidance	1. Pointing accuracy to remembered targets decreased in blur compared to normal vision 2. Pointing accuracy increased when mobility-related cognitive demands were decreased with a physical guide	Design spaces to reduce mobility hazards and the need for safety monitoring
Rand et al. (2019)	Blur P–R = 0.76 logMAR = 1.44 (20/562)	Real-world estimates of distance traveled and speed of self-motion	Vision condition (Blur vs. normal vision)	1. Distance traveled was overestimated in blur 2. Movement speed perceived to be faster in blur	Make walkway ends and walls highly salient
Yamamoto and Philbeck (2013)	Simulated FOV of 3°, control FOV 107° × 86°	Stationary spatial learning task	Vision condition (narrow vs. wide FOV)	1. Spatial layout memory impaired when FOV restricted 2. Impairment was attributed to reduced eye movements	Locate relevant informational objects near each other

*P–R* Pelli–Robson contrast sensitivity value, *LV* low vision, *Blur* simulated reduced acuity and contrast sensitivity, Snellen values are in parentheses

**Fig. 9:**
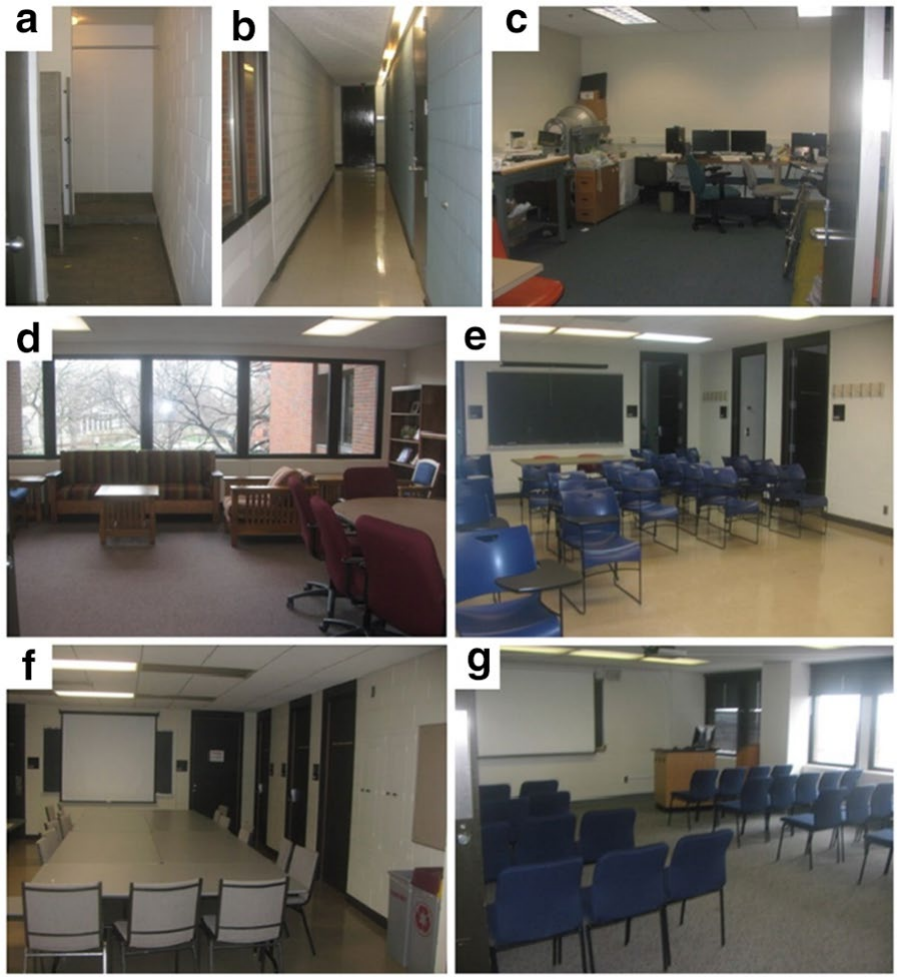
Rooms used in Legge et al. (2016a). Licensed by Creative Commons 4.0

Legge et al. (2016a, b) measured the impact of low vision on both distance and direction estimates in a simple spatial updating task using a three-segment path completion task in seven different sized rooms (see Fig. 9). Surprisingly, none of the reduced vision conditions impaired distance estimates compared to normal vision, but severe blur impaired direction estimates. The automatically acquired information about self-location from real walking (Rieser 1989) may have been sufficient for accurate spatial updating except in the severely blurred vision. In other works, a comparison of spatial updating performance between blind, low vision, and normally sighted age-matched controls showed a surprising lack of difference between groups, suggesting that vision was not necessary for accurate performance in a simple spatial updating situation (Legge et al. (2016a, b). Non-visual (body-based) cues (vestibular, proprioceptive) may be used by individuals with both simulated and natural low vision, which allow for overall accurate performance in spatial updating. However, this spatial updating paradigm was relatively simple, requiring participants to process only three distance segments and two turns. Theories of leaky integration assert that increases in distance traveled and number of turns result in greater error accumulation (Lappe et al. 2007). While normally sighted individuals can use landmarks to “reset” their path integration when it accumulates error (e.g., Zhao and Warren 2015), this capability may not be available to individuals with low vision who do not have access to visual landmarks in the same way, especially in cases of severe acuity or field restriction. Effects of low vision on navigation may thus be more apparent in more complex navigation tasks (longer distances, more turns) that include more opportunity for error accumulation. Rand et al. (2015) tested spaces on the scale referred to as *environmental* space (Montello 1993), which require greater interaction to represent and cannot be experienced from a single location of the observer. These experiments compared spatial memory accuracy for individuals with simulated acuity and contrast sensitivity degradation after navigating through a large indoor building to those individuals’ own performance with normal vision. Memory for the location of landmarks pointed out along the path was worse in the blurred vision condition compared to the normal vision condition. Using a similar paradigm, decrements in memory accuracy were shown when restricting peripheral field of view (FOV), but only when restricted to severe levels around 4° (Barhorst-Cates et al. (2016).

To explain these deficits in performance on spatial cognition tasks with simulated low vision, several studies have tested hypotheses related to perception (Fortenbaugh et al. 2007, 2008; Legge et al. 2016a, b; Rand et al. 2019), attentional demands (Rand et al. 2015), and environmental complexity (Barhorst-Cates et al. 2019). There is some support for perceptual distortions that could influence more global spatial tasks. For example, participants with simulations of severe acuity reduction and restricted peripheral field misperceive the size of the rooms they are in (Legge 2016a, b). Room size estimates might be impaired because of difficulty in perceiving the wall-floor boundary, as seen in Rand et al. (2011). Severe blur results in reductions in visibility of the wall-floor boundary and restricted FOV require a viewer to use more head or eye movements (Yamamoto and Philbeck 2013) to perceive the relationship between the wall and the floor, influencing automatic estimates of angle of declination between line of sight and the wall-floor boundary. But surprisingly, actual low vision and normally sighted subjects showed no difference in room size estimates, in contrast to blind individuals who performed at near-chance levels (Legge et al. 2016b). The discrepant results in simulated compared to actual low vision individuals may be explained by the greater severity of vision reduction in the simulated groups or compensatory perceptual strategies in those with visual impairment (Rieser et al. 1992). Another perceptual explanation is that observers misperceive distance traveled while navigating with visual impairment. A series of experiments by Rand et al. (2019) supports this idea, showing that severe blur results in overestimation of distance traveled and increases the perception of speed of self-motion. Restricted FOV also impairs distance estimates, often resulting in underestimation (Fortenbaugh et al. 2007, 2008).

Beyond explanations based on perception, low vision could influence the cognitive resources needed for spatial learning while navigating. Rand et al. (2015) provided evidence for an account of *mobility monitoring*, which posits that attentional demands from locomotion detract from cognitive resources that could be devoted to spatial learning. They implemented a condition that was designed to reduce cognitive demand associated with safe walking by having the experimenter guide the participant and found better memory compared to an unguided condition, both with severe blur. Further, performance on a concurrent auditory reaction time task was faster while guided, indicating reduced cognitive load, and participants reported less anxiety in the guided condition. These data suggested that mobility-related attentional demands influence spatial learning during low vision navigation, beyond the influence of the visual deficit itself. This is an important finding considering the prevalence of mobility deficits in low vision (Marron and Bailey 1982). Reducing mobility demands can allow more cognitive resources to be devoted to spatial learning. This effect was replicated in an older adult sample, showing an even stronger effect of guidance on improving spatial memory (Barhorst-Cates et al. (2017)). Mobility is more attentionally demanding for older adults even with normal vision (for a review, see Li and Lindenberger 2002), and these data suggest that mobility challenges combined with added attentional demands of low vision may be particularly deleterious for spatial memory in older adults. Effects of attentional demands also extend to navigating with restricted FOV (Barhorst-Cates et al. 2016), where attentional demands increase at moderate levels of FOV restriction.

Recent studies with restricted FOV during spatial learning have tested the impact of active navigation and active search (e.g., looking for named targets at uncertain locations) for targets (Barhorst-Cates et al. 2020) and environmental complexity (Barhorst-Cates et al. 2019). In a comparison of walking and wheelchair locomotion with 10° FOV, spatial memory performance was similar, suggesting that proprioceptive feedback from walking itself does not aid spatial learning (see also Legge et al. 2016a). A possible explanation is the significant mobility challenges faced with restricted FOV locomotion (Jansen et al. 2010, 2011; Turano et al. 2004). While spatial learning could have been facilitated by walking (see Chrastil and Warren 2013), being pushed in a wheelchair may also have facilitated learning by reducing the attentional demands associated with low vision mobility, leading to equivalent performance in the two conditions. Attentional demands were also found to increase with restricted FOV when active search for targets was required, although there were not detrimental effects on spatial memory. However, there may be a critical role for environmental complexity (e.g., more clutter, irregularity in structure) in effects on spatial memory when navigating with restricted FOV. The above-described studies all took place in a campus building with long hallways, with 3–4 turn paths. In contrast, indoor navigation often occurs in less structured, more complex contexts that require more turns in open spaces, such as a hotel lobby or convention center. A study addressed this question of environmental regularity using a museum setting, finding decreased memory and increased attentional load with a less severe 10° FOV restriction (Barhorst-Cates et al. 2019). Open environments, like museums, introduce mobility and visual complexity demands that pose unique challenges to navigation with restricted FOV, more so than environments with structured hallways, where spatial learning during navigation is largely unimpaired except at extreme FOV restrictions.

## Implications for visually accessible architectural design

We conceptualize *visual* accessibility as parallel to the well-established notion of *physical* accessibility. Architects are required by law to comply with accessibility guidelines put forward by the Americans with Disabilities Act (ADA), which primarily focuses on providing physical access for those with physical disabilities, such as the inclusion of elevators and ramps and modification of paths and entrances. The ADA does also include guidelines addressing sensory abilities, but these are primarily focused on signage (e.g., the inclusion of Braille) and other forms of communication. In visual accessibility, we emphasize how vision is used to travel safely through environmental spaces, to perceive environmental features, to update one’s position in the environment, and to learn the layout of spaces. Both physical and visual accessibility closely relate to the Principles of Universal Design for architecture—that the key features of environmental spaces that support its function and mobility should be useful to all people (Mace 1985). Steinfeld and Maisel’s (2012) updated definition of Universal Design emphasizes the *process* “that enables and empowers a diverse population by improving human performance, health and wellness, and social participation”. This revised view acknowledges that designs might not meet all needs, but states that the process brings designs closer to including the needs of as many people as possible. Even though design for visual accessibility focuses on the use of vision (which may not include people who are completely blind), it is an example of this process.

Why is it difficult to take perceptual and cognitive factors into account when designing spaces to enhance accessibility for people with low vision? One reason is that the preponderance of research in the field of architecture is focused on “how buildings are built” corresponding to the second half of the architecture design process, i.e., construction, materiality, and building systems, that have led to innovative and provocative spaces such as Frank Gehry’s Guggenheim Museum Bilbao. Some of these design decisions can unintentionally compromise visibility for low vision, such as creating low-contrast features or glare from skylights or other glass exteriors. While architects are trained to address the challenge of balancing many factors from aesthetics to sustainability to function, some design decisions may unknowingly affect visual accessibility. In contrast, research informing the first half of the architecture design process corresponding to “what is built” has received less attention until recently (Chong et al. 2010). There are exciting movements in architecture that take a human-centered approach to design for human health and well-being, such as the WELL Building Standard (https://www.wellcertified.com/) and Fitwel (https://www.fitwel.org/), as well as academic cross-disciplinary fields focused on the human within spaces, such as the Academy of Neuroscience for Architecture (http://www.anfarch.org/) and the emerging area of Human-Building Interaction (e.g., https://www.intelligentenvironments.usc.edu/). These movements draw on and extend work of the interdisciplinary field of Environmental Psychology begun over 50 years ago (Canter and Craik 1981; Craik 1973). Progress toward universal design supporting the functions of built spaces can be seen in the example of the useful set of design guidelines for built environments put forward by the Low Vision Design Committee of the National Institute of Building Sciences (NIBS) in 2015 (https://www.nibs.org/page/lvdc_guidelines) and lighting guidelines put forward by the Illuminating Engineering Society (Barker et al. 2016). A number of the NIBS guidelines relate to the ideas of visual accessibility and the perception of local and global features for spatial behavior and could be informed by basic science approaches such as the methods described above. For example, the guidelines suggest avoiding patterns on flooring that could be mistaken for steps and placing ottomans or tables that are low or have transparent parts. The basic research described here establishes a scientific foundation for more general and future guidance in these directions.

Together, the body of work on perceiving local and global features in low vision contexts provides some initial insights and recommendations for architectural design that can enhance visual accessibility. These are summarized in Tables 1 and 2. Beginning with the basic features supporting travel through spaces such as sidewalks, corridors, and stairways, research has identified challenges that could inform design. The “ramps and steps” work identified that *enhancing the contrast at step transitions with directional lighting* helped detection, but that providing high contrast texture on these surfaces hurt detection. The research also shows that while the subtle image cues of discontinuities in edge contours are very susceptible to changes in viewing conditions, cues that are less dependent on acuity facilitate perception of these environmental features. One good example is the *cue of height in the picture plane* for the identification of ramps, which was useful in blurred viewing conditions even at relatively shallow ramps. For perception of absolute scale that informs localization of these features, the *visual horizon combined with eye height* is readily used even in severely blurred viewing conditions. Low vision distance perception studies showed that even when viewers could just barely detect the presence of the object, they relied on vision of the floor-wall boundary to inform distance judgments. This finding is significant, as it suggests that if interior design is such that low contrast (or no contrast as in the black carpet and wall intersection in Fig. 6) impairs the perception of the floor-wall boundary, observers are likely to misperceive spatial locations and possibly room size as well. These examples along with empirical work emphasize the importance of *high contrast at the floor-wall boundary*. Research on objects as hazards supports some of the initial guidelines from the NIBS about visibility of features in terms of size and placement of environmental objects such as signs, poles, or furniture. For example, the *visibility of object-ground contact* matters. One study showed quantitatively that when viewers could no longer detect the object’s attachment to the ground, they perceived the object to be at a different location. Broadly for detection of objects, contrast matters for visibility with blurred vision, but more subtly, the *contrast between object and background is dependent on lighting arrangement*. *Shape of environmental objects* could also be considered, as curved objects were generally more visible than straight-edged objects under blur viewing conditions. Finally, an *object’s angular size* could be taken into account in the design of paths for pedestrians.

Basic research on perception of global features used to support spatial updating and spatial learning is in some ways consistent with the focus on local features summarized above. Those with simulated or actual low vision show relatively intact abilities to judge room size and update self-location after traversing simple paths within vista scale spaces, unless under extreme acuity/contrast sensitivity degradation. This is likely because of the ability to use *salient wall-floor boundaries* as well as *non-visual body-based information for spatial updating*. Blur does influence dynamic perception of distance traveled which may contribute to errors in learning of spatial layout while navigating. In environmental-scale navigation tasks, we have identified consistent effects of increased attentional demands for mobility associated with decreased accuracy for remembered locations. This occurs with both reduced acuity and contrast sensitivity and severely reduced peripheral field. While these are very different visual deficits, they both impact the automaticity of walking and show that designers should *consider the associated cognitive factors that accompany the complex interaction of visual parameters*. Navigating with visual impairment involves constant spatial problem solving (Giudice 2018) and associated increased anxiety about travel. The findings from the museum study (Barhorst-Cates et al. 2020) suggest that more complex environments and navigation paths may raise different issues in visual accessibility. Possibilities for reducing cognitive demands during travel might be to ensure unobstructed corridors and walkways and consider the impact of placement of highly visible landmarks and signs that could be used from a distance.

From a theoretical perspective, the research on global spatial features also suggests that non-visual spatial information can be used to solve navigation tasks (Giudice 2018). Loomis et al. (2013) propose an “amodal hypothesis” that accounts for functional equivalence, or similar behavioral performance in spatial tasks regardless of the sensory channels through which spatial information is conveyed. A body of research suggests that in many circumstances we act similarly in spaces that are conveyed by haptic stimuli, auditory stimuli, spatial language, or by vision. However, when designing for visual accessibility, it is important to consider the increased uncertainty that comes with reliance on degraded visual information that parallels what is known for use with non-visual information. For example, haptic perception can provide information about potential obstacles and distances, but only within the range that can be reached with the arm or long cane. Auditory perception provides cues for locations of objects at greater distances, but is less precise in specifying distance, direction, and self-motion (Giudice 2018). Similarly, low vision navigators with reduced acuity and/or contrast sensitivity also experience uncertainty in the available visual information and this uncertainty increases dramatically with the greater distances and complexity of spatial problem solving inherent in acting over larger-scale environments.

While the basic research has provided some support for the NIBS design recommendations for low vision, guidelines or intuitive practices can only take us so far toward the goal of visual accessibility. As noted throughout, there is variability in performance across spatial scenarios because of the difficulty in predicting the complex interaction between lighting conditions, environmental geometry, surface materials, and visual deficits. It is important to note that architects do not purposely design in ways that would exclude any population of users. Most often, if there are problematic spaces, it is reflective of lack of knowledge to the specific issues of those select populations. With the multitude of considerations that architects must integrate into the design (e.g., building program/function, structure, building systems, codes, zoning), moving to Universal Design through the consideration of low vision issues is a challenge.

## Future directions for designing visually accessible spaces

Basic research in low vision perception identifies both capabilities and limitations associated with spatial cognition and navigation in visually restricted contexts. There are still many open questions as to the influence of type and severity of vision loss on the functional capabilities underlying independent travel. A future goal should be to test a wide range of low vision individuals on the types of paradigms that have been developed. This would serve to generalize beyond simulated low vision by varying the extent of visual impairment in ways that naturally occur with age or eye disease as well as account for the role of experience and strategies that people with low vision have. Notably, the “blur” created with restricted viewing goggles in many of the studies reduced acuity and contrast sensitivity together in ways that are not necessarily representative of specific forms of low vision. The simulations also independently limited acuity/contrast sensitivity or visual field loss, while many people with low vision experience both types of deficits together. Thus, there are clear benefits to expanding empirical work to include the diversity of low vision conditions in research on visual accessibility.

As we described earlier, the prevalence of low vision is growing worldwide, and the health and well-being of this population depends on the ability to have access to spaces in ways that promote independent travel. Future work in the design of visually accessible spaces must consider that visual impairment does not exist in isolation from other health problems. The prevalence of many eye diseases (e.g., age-related macular degeneration, glaucoma) is highly correlated with age, and there is evidence for comorbidities with cognitive impairments, hearing impairments, and depression (Whitson et al. 2011). Other comorbidities exist with physical disabilities such as the peripheral neuropathies associated with diabetes-related visual impairment (Tesfaye et al. 2010) or the increased likelihood of requiring a walker or wheelchair with age. Future directions of research should consider the diversity and individual differences inherent in a population with low vision.

There is potential in new assistive technologies that could supplement visually accessible design and facilitate the space perception and spatial cognition needed for safe and efficient navigation. However, the development of these technologies requires a human-centered design approach (O'Modhrain et al. 2015) that considers realistic scenarios and usability of visually impaired users—an approach that is not always typical of the designers (Giudice 2018). Furthermore, effective design of assistive technologies needs to be informed by an understanding of the perceptual and cognitive processes that underlie spatial representation and navigation (Giudice 2018; Loomis et al. 2012). For tasks that we define here as relying on global features, such as spatial updating and navigation along more complex routes, speech-enabled GPS-based navigation devices may be used to provide information about spatial layout, position, and orientation information. These systems currently work best outdoors, and assistive technology still needs to be developed for indoor wayfinding (Giudice 2018; Legge et al. 2013). An important consideration for the use of any type of assistive device is the additional cognitive processing required. As described in the spatial learning studies reviewed here, navigation with restricted viewing is inherently more cognitively demanding. The additional cognitive load required for use of an assistive technology could negate its positive effects. Future work is needed to understand the multisensory spatial information that is used in complex wayfinding and navigation tasks so that it can be conveyed and used effectively.

## Data Availability

Not applicable.

## Abbreviation

FOV: Field of view

## Footnotes

1 Acuity is the ability to detect fine-scale patterns and is often clinically measured in terms of LogMAR (Bailey-Lovie chart) which is the logarithm of the minimum angle of resolution (Bailey and Lovie-Kitchin 2013). A logMAR value of 0 indicates normal acuity (20/20 Snellen), and larger values correspond to lower acuity (logMAR 1.0 = 20/200 Snellen). In this paper, we report both logMAR and Snellen values. Increases in the denominator of the Snellen fraction correspond to decreases in acuity. The Pelli–Robson Contrast Sensitivity chart (Pelli et al. 1988) measures contrast sensitivity (the ability to see small changes in luminance) as the threshold lowest contrast for letter recognition of black/gray letters on a white background. A value of 2.0 is normal contrast sensitivity, and the value decreases with loss of contrast sensitivity. Field of view is the amount of the environment that is visible at one time and is described in terms of degrees of visual angle.
